# Generative artificial intelligence in otorhinolaryngology: From innovation to public health transformation

**DOI:** 10.1016/j.bjorl.2025.101714

**Published:** 2025-09-11

**Authors:** Alexandre Vallée

**Affiliations:** Foch Hospital, Department of Epidemiology and Public Health, Suresnes, France

Over the past year, Generative Artificial Intelligence (GAI), spearheaded by Large Language Models (LLMs) such as ChatGPT, has transcended its status as a technological curiosity to become a topic of urgent relevance for clinical practice, public health, and medical education. Nowhere is this transformation more visible than in specialties like Otorhinolaryngology (ORL), which combine high diagnostic complexity, large volumes of patient interactions, and increasing demands for personalized care. In this context, ChatGPT and similar models are not merely futuristic tools; they are potentially transformative agents for reimagining how ENT care is delivered, how patients are engaged, and how professionals make informed decisions.

ChatGPT, developed by OpenAI, is based on transformer architecture and trained on vast textual corpora, enabling it to generate coherent, context-sensitive language. Beyond its linguistic fluency, the model is capable of performing Natural Language Understanding (NLU), summarizing medical literature, interpreting clinical notes, and simulating human-like conversations. These capabilities offer immense value in clinical decision support, patient education, triage, and the democratization of access to medical knowledge, all of which are particularly relevant to ORL, a field with both high patient volume and complex symptomatology.^1^

The clinical and public health implications of such tools are profound. For instance, ChatGPT may serve as an intermediary for patients presenting with non-urgent Ear, Nose, and Throat (ENT) symptoms such as nasal congestion, sore throat, tinnitus, or snoring, symptoms that often lead to inappropriate emergency consultations or delayed care. By providing accurate, real-time, and empathetic responses, generative AI tools could help triage symptoms, guide patients toward appropriate care, and reduce unnecessary healthcare utilization.^2^

On a broader scale, GAI can be a catalyst for improving health literacy. ENT conditions are often misunderstood or stigmatized, particularly in the domains of voice, hearing, or sleep disorders. With proper safeguards, ChatGPT could deliver tailored, culturally sensitive health information that empowers patients, especially in low-resource settings or underserved populations where access to ENT specialists is limited.^3^

The potential does not stop at the patient interface. For clinicians, ChatGPT offers the promise of enhancing diagnostic reasoning by integrating vast bodies of medical knowledge, previous case data, and clinical guidelines into a user-friendly, conversational interface. In the setting of sialendoscopy, for example, AI could help interpret imaging data, suggest relevant differential diagnoses, and support pre-procedure counseling.^2^ Similarly, in surgical planning or perioperative care, ChatGPT could assist in automating informed consent generation, documentation, and access to real-time procedural checklists, although these applications remain experimental.^4^

Educationally, ChatGPT has already demonstrated utility in exam preparation for otolaryngology board certifications. It can generate practice questions, explain correct and incorrect answers, and adapt explanations to the learner’s level. This may prove especially valuable for training programs in low-income countries, where access to expert faculty and updated educational materials is limited. The impact on continuous medical education, particularly in rapidly evolving fields like rhinology or head and neck oncology, could be substantial.

However, this enthusiasm must be tempered by critical evaluation. One of the most pressing concerns is the risk of misinformation. ChatGPT, like other LLMs, is prone to “hallucinations”, plausible but incorrect answers that can be difficult to detect without expert oversight.^5^ In medicine, where the stakes involve human lives, such errors are unacceptable. Moreover, the model’s training data only extends to 2021, limiting its access to recent guidelines, drug updates, or new clinical evidence.

Even more concerning is the risk of perpetuating health inequities. AI systems often reflect the biases embedded in their training data. Without intentional correction, ChatGPT could produce biased outputs regarding gender, race, disability, or socioeconomic status, with harmful implications for diagnosis, treatment access, and health communication. In the ORL domain, this could exacerbate existing disparities in cochlear implant candidacy, HPV vaccination uptake, or screening for head and neck cancers.^5^

Privacy and data security represent additional barriers to adoption. For ChatGPT to be safely integrated into Electronic Health Records (EHRs) or patient-facing apps, stringent encryption, traceability, and de-identification protocols must be in place. Moreover, data governance must ensure that patient consent is fully informed, revocable, and compliant with evolving legal frameworks such as GDPR or HIPAA equivalents in Latin America and elsewhere.^4^

To address these limitations, the responsible deployment of generative AI must be governed by a robust framework of ethical, technical, and clinical safeguards. Human oversight is paramount. AI outputs must be interpreted by qualified professionals, and clearly marked as non-authoritative when presented to patients. Continuous performance monitoring, using predefined metrics for accuracy, bias, relevance, and user satisfaction, should become standard practice in ORL clinics that integrate ChatGPT.^4^

Additionally, integration with clinical workflows must be seamless. This includes developing APIs and user interfaces that can securely connect ChatGPT to ENT-specific software, EHR systems, and diagnostic devices. Pilot programs in academic hospitals could serve as sandboxes to test, refine, and validate such integrations before large-scale rollouts.

Interestingly, there is also potential for AI to augment mathematical reasoning in clinical epidemiology and health services research. Recent work has shown how machine learning can guide human intuition in uncovering latent patterns between variables, an approach that could be leveraged to explore correlations between ENT symptom profiles, environmental exposures, and treatment outcomes. This further strengthens the case for GAI as a tool not just for communication, but for discovery.

Ultimately, ChatGPT should be viewed not as a substitute for clinical judgment, but as a collaborative instrument, one that enhances the clinician’s ability to deliver informed, compassionate, and personalized care. The goal is not to replace otorhinolaryngologists, but to support them in navigating an increasingly complex healthcare environment, marked by time constraints, information overload, and growing patient expectations.

From a public health standpoint, the integration of GAI into otorhinolaryngology aligns with broader efforts to build more resilient, accessible, and participatory healthcare systems. By facilitating early diagnosis, optimizing triage, supporting education, and enhancing engagement, tools like ChatGPT may ultimately reduce the burden of ENT diseases, particularly in vulnerable populations.^3^ However, success will require interdisciplinary collaboration, between clinicians, AI developers, ethicists, patients, and policymakers, to ensure that these technologies are implemented not only efficiently, but equitably.

In conclusion, generative AI represents a powerful ally in the evolution of otorhinolaryngology. But its promise will only be realized if we proceed with humility, vigilance, and a steadfast commitment to human-centered care. The future of ENT may be written, in part, by machines, but the responsibility will always rest with us.

## ORCID ID

Alexandre Vallée: 0000-0001-9158-4467

## Ethical considerations

Not applicable.

## Funding

This research received no external funding.

## Data availability statement

Not applicable.

## Declaration of competing interest

The author declares no conflicts of interest.
